# New York State Veterinary Medical Society

**Published:** 1895-11

**Authors:** Nelson P. Hinkley

**Affiliations:** Secretary


### SOCIETY PROCEEDINGS.

### NEW YORK STATE VETERINARY MEDICAL SOCIETY.

THE sixth annual meeting was held at the lecture rooms of the New York College of Veterinary Surgeons, New York City, September 5 and 6, 1895.

FIRST DAY, THURSDAY, SEPTEMBER 5, 1895.

The meeting convened at 11 A.M. The call of the roll showed the following members present: Drs. R. D. Austin, W. L. Baker, R. R. Bell, J. M. Currie, W. S. Corlis, G. G. Dean, G. E. Gangloff, Wilson Huff, J. A Bell, R. S. Huidekoper, George H. Berns, A. W. Baker, Charles Cowie, A. Crowforth, J. A. Genung, Thomas Giffin, N. P. Hinkley, J. A. Huhne, W. H. Kelley, C. D. Morris, L. McLean, William Machan, F. Morrow, Arthur O'Shea, S. Sommerville, Jr., J. P. Thompson, John Wende, Lester E. Willyoung, W. J. Wadsworth, A. G. Wicks, E. D. Ingalls, Prof. James Law.

The minutes of the fifth annual meeting were read and adopted. The minutes of the special meeting, held June 11, 1895, weie read. A motion was made by Dr. L. McLean and seconded by Dr. G. H. Berns that the minutes of the special meeting be not accepted, but be rescinded. Remarks were made on this question by Drs. Baker, Crowforth, Kelley, Morris, Law, and others. Dr. Crowforth offered an amendment to Dr. McLean's motion that the minutes of the proceedings of the special meeting of June 11, 1895, be approved and stand as read. This amendment was seconded by Dr. W. L. Baker; was put to a standing vote, with the following results: For the amendment, 18; against the amendment, 5. The proceedings of the special meeting was ordered spread on the minutes. The President, Dr. Rush S. Huidekoper, then read his address,[1] which was received with applause.

The Secretary-Treasurer, Dr. Nelson P. Hinkley, read his report for the past year, as follows:

"In submitting for your consideration the past year's work of this office, I do so with satisfaction and pleasure. The condition of this Society to-day is one every member should feel proud of. Six years ago when you saw fit to honor me with the position of Secretary, I accepted the trust with pleasure, and since then you have through your votes at each annual meeting retained me in office.

"In the meantime our membership-roll has increased and multiplied so rapidly that it numbers over one hundred members, with more asking for admission at each meeting. As you are all aware, the past six years, in the history of this Society, have been six years of labor and watching, by every active member in our oganization, in our endeavors to better and elevate our profession, by procuring laws that would lift the veterinary profession in this State from the rut of ignorance, charlatanism, quackery, and from the public abuse she so long has suffered. How we succeeded can best be judged by a comparison of the laws then and now.

[1] Will appear in December number.

"Each succeeding year has brought with it new and increased work for this office. Slowly but surely the business of this office has increased, until to-day it is of such a magnitude that none but one who has had the responsibility and execution of this work can comprehend the time and labor required to perform its requirements faithfully and efficiently. You will better understand the amount of work done during the past year by some figures relative thereto. There has been 1097 communications received at this office, regarding matters pertaining to this Society, nearly all of which required an answer; 1309 type-written letters were sent from this office to members of this Society and other veterinarians relative to Society matters; 500 printed forms of proposed laws, 100 notices of special meeting, 704 programmes, 704 notices of annual meeting, 704 notices of recent law, 704 notices to members contemplating attending the United States meeting, 154 notices to delinquent members, and 97 bills for annual dues; making a total of 4116 communications that have been written out and mailed. This work has occupied a large share of my personal time both day and night, and then only accomplished by the aid of my private clerk and stenographer.

"In addition to this I have had printed and bound 500 copies of our Constitution and By-Laws, as revised and adopted by the Society, October 11 and 12, 1893. I have also procured from nearly every county clerk in this State a complete copy of the names, location, etc., of every veterinarian registered in his county. This data will in my opinion be found to be very useful whenever the members of this Society see fit to have compiled and printed a veterinary blue-book or registry of this State.

"As your Secretary I have visited Albany to appear before the Committee of Public Health with the other members of the Legislative Committee in behalf of the recent law pertaining to the regulation of veterinary practice in this State. I have endeavored by letters and personal interviews with several different members of this Society, located in different counties to encourage them to form county veterinary societies, but up to the present writing I know of only three or four having been formed. This I think is a great mistake the veterinarians are making by not forming these societies, as there is not a county in this State but could organize a society that would be a credit to its promoters. It would not only benefit the members by the reading and discussion of papers, communication of cases, etc., but the public would more readily recognize the members as progressive and enterprising in their profession, and deserving of their fullest confidence and trust, for in this way the members could soon procure for themselves the much-needed reform in the way of having qualified veterinarians appointed by our health boards as milk, dairy, and meat inspectors, as well as city veterinarians to our fire, police, and public departments. These county societies should be closely allied and in touch with the State Society in all her workings. They should at all times be on the alert and carefully watch the public offices in which the laws and ordinances are framed and executed that would in any way benefit or injure our profession. They should always be ready to assist in everything that will benefit our profession, our clients, and the public at large. The members of these societies should make their individual workings as professional men and as honest, upright citizens so felt in the county in which they reside that their services, advice, counsel, and company would always be desirable. Through their Secretary they should make annual reports of their work and of the character and frequency of disease as found and treated by the members, and the results; the stamping out of contagious and infectious diseases of animals; the improvement in the sanitary conditions of our animals, our dairies, abattoirs, markets, etc., should be matters that these societies should not only interest themselves in but be the leaders and investigators. Committees should at all times watch all Legislative matters carefully. In this way the State Society would at all times have at its command an intelligent and comprehensive compendium of all affairs pertaining to the veterinary art in this State. I trust that you will take such action at this meeting as to warrant a systematic method for organizing county societies.

"The finances of this Society at present are in fair condition. Of course as the members increase in numbers, so the work of the Society increases, and the same can be said of the expenses of running the Society. Right here I would like to impress on the members the necessity of prompt payment of their dues, initiation fees, and assessments, in order that the Society's affairs can be conducted on business principles and with business methods. This office, as my report will show, has carried the Society's obligations for the past three years, and I have been obliged to expend from $100 to $200 per year out of my own personal funds to insure the proper and successful working of the Society. I earnestly urge that such means and ways may be adopted and put into effect at this meeting that will place this Society out of debt and leave a balance in the treasury. Surely there is not a member who has not received sufficient benefit from this Society to be liberal in his contribution for its support. Personally I have sacrificed a good share of my time and some money for its advancement, and am willing to continue to do so, but not in the capacity of Secretary, as I feel as if six years of hard work should be my share, considering that the work of organizing is complete, and also that in this Society we have so many members who by natural ability and individual circumstances would fulfil and carry out the workings of this office to the point of perfection and continued success that has been my aim and desire during my term of office.

"In conclusion, fellow-members, I wish to thank you all for your generous support and aid, and your oft-repeated assurance of good will, which I most heartily appreciate. I wish also to thank the Editors of the JOURNAL OF COMPARATIVE MEDICINE AND VETERINARY ARCHIVES for their ever-kind and generous treatment to the frequent demands this Society has made on their valuable journal for space for our Society matters, and I trust that every member of this Society will ever remember the kind treatment we have received from their hands and always show their appreciation of the same by a generous support.

"In closing the report of this office, I do so with a feeling of relief for the arduous and trying position, but, as I mentioned in the first part of my report, it is with satisfaction of a conscientious effort on my part to have discharged the many responsibilities that it involved."

A motion was made by Dr. Morrow, seconded by Dr. Baker, that the Secretary's report be received and adopted as read and the suggestions therein discussed; carried.

The Chairman of the Board of Censors, Dr. Claude D. Morris, reported that twenty-five applications for membership had been received, and that the board asked for a recess to consider the applications. On motion of Dr.

W. H. Kelley, seconded by Dr. Wilson Huff, the Board of Censors were allowed further time to examine the credentials of the new applicants for membership.

Dr. Thomas Giffin, of New York, invited the members of the Society to a lunch, which was tendered by the New York County Veterinary Medical Society. The invitation was accepted, and on motion of Dr. O'Shea, seconded by Dr. Austin, the meeting adjourned for lunch, to convene again at 2 P.M.

### Afternoon Session, Thursday, September 5, 1895.

The Board of Censors announced that they would consider the applications of those intending to become members of this Society and report Friday morning.

The report of the Committee on Prosecution, Dr. John Wende, Chairman, was next read. Regarding test-cases in Erie County, Dr. Wende read a letter from Dr. Spencer, of Dunkirk, in which he asks for advice as to the prosecution of quacks. Dr. Wende answered his letter, telling him to bring an action against the offender or call the attention of the district attorney to the case. Dr. Wende also read a letter from Dr. Carpenter, of Johnstown, in which he requests the chairman to write to Charles Mansfield, of Johnstown, and one Clement, of Gloversville, to the effect that they are practising illegally, not being registered; and another to J. G. Sillico, of Gloversville, not registered. Dr. Wende stated that he had written to each, accordingly, in his official capacity. Had received in answer letters from two lawyers. One letter, regarding Mansfield, he read, which set forth that Mansfield had been in practice since 1865; had complied with all the requirements, and that in his opinion the present law does not affect him. Mansfield also sent testimonials as to his ability, etc., in which allusion is made to a proprietary medicine, "the horse's friend," "best cough and heave-powder known," "liniment without equal," "expert trainer and educator of colts," etc., duly vouched for and signed by would-be reputable citizens. District attorney wrote to Dr. Wende regarding Lucas Clement, of Gloversville, stating he began practice forty years ago; was registered in Montgomery County, but later moved to Fulton County, registering in one county and practising in another. District attorney asked for instruction; also gave his opinion that the present law did not effect Clement. Dr. Wende, up to the present time, had not answered this letter. Dr. Kelley stated that, since this, Clement has obtained his transcript and sent it to the Board of Regents, properly signed, thus making him legally registered in Fulton County.

A discussion followed the report of the Prosecuting Committee, and Dr. Morris proposed that another committee be appointed to look after report of cases. Dr. Kelley said "that the Board of Regents advises the same, but to proceed slowly in all cases until after the first examination is held." It was also suggested that the registration-book of each county be examined every six months. Dr. Law stated that in his opinion most offenders are such through ignorance of the law, and to renovate it by giving each offender an opportunity to rectify themselves by coming before the examiners or by obtaining a transcript. Dr. McLean advised not to be too lenient in such cases, and that they should be dealt with summarily. Dr. Morris cited an instance where a veterinarian presented his diploma to the county clerk of Putnam County and was told that being registered in one county it was not necessary to do so in another. Dr. Berns moved, seconded by Dr. McLean, that the report of the Prosecuting Committee be approved; carried.

The Secretary called the attention of the meeting to delinquent members; stated that some are three, four, and five years behind in their dues. Moved by Dr. Morris, seconded by Dr. Kelley, that the names of these delinquents be read, the list numbering thirty-seven. Moved by Cowie and seconded by Dr. Kelley that each one be dealt with separately and suspended; the first name on the list being A. W. Baker, of Cortland, and a satisfactory explanation was furnished in this case, Dr. Baker's name was not stricken from the roll. The following names were then (by separate vote of the house) dropped from the roll and suspended for non-payment of dues: Drs. H. B. Crandall, W. A. Conklin, A. Drinkwater, W. G. Dodds, B. Howes, A. L. Hunter, J. G. Hill, A. H. Ide, J. P. Jeffrey, H. C. Klicker, W. E. Langford, J. G. Lillico, G. H. Moulter, L. G. Moore, H. D. Martin, E. J. McLeod, L. E. Palmer, Emil Pohl, G. H. Roberts, F. A. Rich, D. K. Seltzer, H. Sutterby, F. Sutterby, W. L. Stevenson, G. H. Summerfeldt, P. K. Sidebottom, W. B. Switzer, W. W. Woolston, C. J. Willgansz, J. Whytock, F. E. Williams, J. D. Whyte, E. A. Wieland, D. C. Papworth, A. Dowswell.

A communication was read from Dr. Wadsworth, of Seneca Falls, in which it was shown that a patent-medicine testimonial was signed and indorsed by Dr. J. M. Chase, of that place. The matter was referred to the Board of Censors for investigation.

Moved that Article 5 of the by-laws shall be altered to read: "It shall be the duty of the censors to take proof of the credentials," etc.

*New Business.* The matter of a blue-book, containing names and residences of all practitioners of this State, was brought before the meeting. The Secretary explained its object and merits. It was discussed pro and con and left with the officers of society.

Dr. Kelley next explained the recent law pertaining to veterinary practice and medicine.

Dr. Baker next proposed an amendment to Article 3 of the Constitution, or to alter it to read as follows: "This Society shall consist of legally-registered veterinarians residing in the State of New York, who shall be elected after being properly vouched for and approved by the Board of Censors." Signed, W. L. Baker and G. H. Berns. This motion was seconded by Dr. Giffin. It was, however, objected to by Dr. McLean. The matter was held for our next annual meeting, when it will be voted on by the members present.

The question arose what shall be done with certificates of membership held by suspended members. It was moved by Dr. McLean and seconded by Dr. Huff that the names of suspended members be published in local papers in their respective vicinities, but the general sentiment prevailed that this procedure would be wrong; the motion was not carried. It was moved by Dr. Crowforth, seconded by Dr. Austin, that the Secretary notify each and every member suspended to return his certificate; carried.

It was moved by Dr. Hinkley, seconded by Dr. McLean, that a vote of thanks be tendered the Hon. Mr. Holmes, Senators Cantor and Sullivan, and Dr. O'Shea for their efforts and success in urging the passage of the recent bill through the Legislature and obtaining the present law; carried.

It was moved by Dr. Berns and seconded by Dr. Austin that also a vote of thanks be tendered the Legislative Committee of this Society; carried.

Next Drs. Hinkley, Kelley, and Huidekoper were elected delegates to the meeting of the U. S. V. M. Association, to be held at Des Moines, Iowa, September 10 and 11, 1895.

The Treasurer's report was next read and approved of, after which came the reading of papers. A paper on "Acaro Demoiex Folliculorum" was read by Dr. Crowforth, of Lockport, and "Enteralgia" by Dr. Willyoung,[1] of Buffalo. A lively and interesting discussion followed the reading of the papers. Dr. Berns stated that he prevented in a measure the development of subcutaneous abscesses after the use of the trocar by using injections of carbolic solutions. Dr. Neher usually applies a mild blister to the parts immediately surrounding the point of puncture. He also stated that the stomach may be punctured from an external point, but this was not substantiated. Different opinions were expressed as to the possibility of vomition in horses, some contending that it only occurs after a partial or complete rupture of the stomach, while others claimed that it was a natural physiological function, it being prevented only by the anatomical conformation of the throat. Crib-biting was also discussed at length.

Moved by Dr. Hinkley, seconded by Dr. Kelley, that an adjournment be taken to Friday morning at 10 A.M.; carried.

FRIDAY, 10 A.M., SEPTEMBER 6, 1895.

Roll-call showed thirty-five members present, with several visitors. The Chairman of the Board of Censors, Dr. Claude D. Morris, reported that owing to the incomplete applications that some applicants had made out he requested that the Board be allowed more time to consider the applications. Moved by Dr. Baker and seconded by Dr. Gangloff that the Board of Censors be allowed further time for reconsideration of applicants. At this juncture Dr. Hoskins, President of the United States Veterinary Medical Association, entered the room, and was received with loud applause. It was moved and seconded that the courtesy of the meeting and privilege of the floor be extended to Dr. Hoskins. Dr. Hoskins thanked the members for the courtesy accorded him, and extended a cordial invitation to all the members present to attend the meeting of the U. S. V. M. A. at Des Moines, Iowa.

At 2 P.M. the Board of Censors reported and recommended the following veterinarians for membership in the Society: Drs. M. Knott, William Swan, S. S. Field, J. E. Ryder, C. Doepel, H. Wellner, C. P. Martin, F. W. Turner, J. H. Ferster, T. Sherwood, W. Lellman, R. C. Jones, W. H. Jackson, Robert Dickson, J. L. Ronan, V. Huppe, W. W. Dickey, Thomas O'Dea, Robert W. Ellis, H. Neher, E. B. Ackerman, E. Lalowell. The applications of Drs. Williams and Cady were not acted upon, because not properly vouched for. The application of Dr. Gill was held over until the next meeting. An adjournment here was taken for lunch, which was tendered the Society through the courtesy of the members of the New York County Veterinary Medical Society. After the vouchers were signed by the Board of Censors one ballot was cast by the Secretary for all remaining

[1] See page 721.

candidates, after which the successful members were introduced to the Society.

The next order of business was the nomination of officers. For President there was but one nomination, Dr. N. P. Hinkley; for Vice-President, Drs. Giffin, J. A. Bell; for Secretary-Treasurer, Dr. C. D. Morris; for Censors, Drs. Cowie, O'Shea, Baker, Huidekoper, Law, Wende. As only one nomination each existed for President and Secretary-Treasurer, the Assistant Secretary was authorized to cast one ballot, which was done, electing Dr. N. P. Hinkley as President and Dr. C. D. Morris as Secretary-Treasurer. Balloting for Vice-President was next in order. Dr. Giffin received 22 votes and Dr. Bell 14 votes; Dr. Giffin was declared elected. For Board of Censors, Dr. Baker received 34 votes, Dr. Wende 27, Dr. Law 33, Dr. Huidekoper 28, Dr. O'Shea 34, Dr. Cowie 23; Drs. Baker, Wende, Law, Huidekoper, and O'Shea were declared elected.

It was then moved and seconded that our next place of meeting be at Buffalo; carried. Dr. Hinkley, the newly-elected President, took the chair, made a few remarks thanking the Society for conferring on him this honor of office, etc. Remarks were also made by Drs. Giffin and Morrs. A vote of thanks was next tendered Dr. Morris for his efforts in investigating the charges in the name of the Society, and it was also resolved to reimburse him for expenses during the same. A vote of thanks was tendered the New York College and New York County veterinarians for the use of the rooms and the courteous entertainment shown us during the entire meeting. A vote of thanks was also tendered the retiring President and Secretary-Treasurer.

Dr. Law's amendment to the by-laws, also Dr. Baker's, were put on file and will go into the hands of the Committee on Constitution and By-Laws, both being submitted in writing.

An adjournment was here taken to witness the operations of castration of a ridgling by Dr. John Wende, and arytenectomy by Dr. H. D. Gill.

The meeting convened at 6.30 P.M., when Dr. Law expressed his opinion in the case of Dr. Gill, stating that he was under the impression that Dr. Gill was eligible for membership. Dr. Law moved that the Board of Censors again consider the Gill matter. An amendment to this was moved, seconded, and carried that the matter be laid over until the next regular meeting.

It was moved by Dr. Bell and seconded by Dr. L. E. Willyoung that the meeting adjourn *sine die*. Thus ended one of the best, most interesting, and largest attended meeting ever held since the inauguration of this Society.

DR. NELSON P. HINKLEY,
Secretary.

---

